# Simulation of high-intensity focused ultrasound lesions in presence of boiling

**DOI:** 10.1186/s40349-016-0056-9

**Published:** 2016-03-31

**Authors:** Anthony Grisey, Sylvain Yon, Véronique Letort, Pauline Lafitte

**Affiliations:** Theraclion, 102 Rue Etienne Dolet, Malakoff, 92240 France; CentraleSupélec, Mathematics in Interaction with Computer Science, Grande voie des vignes, Châtenay-Malabry, 92295 France

**Keywords:** HIFU, HITU, Modeling, Thermal model, Nonlinear acoustics, Numerical simulation, Bioheat, Boiling

## Abstract

**Background:**

The lesions induced by high-intensity focused ultrasound (HIFU) thermal ablations are particularly difficult to simulate due to the complexity of the involved phenomena. In particular, boiling has a strong influence on the lesion shape. Thus, it must be accounted for if it happens during the pulses to be modeled. However, no acoustic model enables the simulation of the resulting wave scattering. Therefore, we propose an equivalent model for the heat deposition pattern in the presence of boiling.

**Methods:**

Firstly, the acoustic field is simulated with k-Wave and the heat source term is calculated. Then, a thermal model is designed, including the equivalent model for boiling. It is rigorously calibrated and validated through the use of diversified ex vivo and in vivo data, including usually unexploited data types related to the bubble clouds.

**Results:**

The proposed model enabled to efficiently simulate unitary pulses properties, including the sizes of the lesions, their morphology, the boiling onset time, and the influence of the boiling onset time on the lesions sizes.

**Conclusions:**

In this article, the whole procedure of model design, calibration, and validation is discussed. In addition to depicting the creative use of data, our modeling approach focuses on the understanding of the mechanisms influencing the shape of the lesion. Further work is required to study the influence of the remaining bubble clouds in the context of pulse groups.

## Background

In many industrial domains, numerical simulation has been adopted as a primary tool for optimizing processes and devices. However, the simulation of high-intensity focused ultrasound (HIFU) treatments presents specific challenges. Thus, modeling the lesion creation process remains complex in many clinically relevant situations, where cavitation or boiling occur [1]. In these cases, where the influence of the bubble cloud cannot be directly simulated, equivalent models have to be developed [1, 2]. Beyond their predictive capability, which is by nature restricted, they aim at producing a “useful understanding” [1] by identifying the main physical phenomena shaping the lesion. In this context, this paper describes a rigorous approach for designing, calibrating, and validating an equivalent model to simulate the in situ temperature field.

Acoustic simulation of HIFU beams has been greatly studied during the past decades, and several wave equations have been derived, such as the widely used Khokhlov-Zabolotskaya-Kuznetsov (KZK) [3] equation and the Westervelt equation [4]. The choice of the acoustical model is not discussed in details in this paper, but the interested reader can for example refer to the book of Hamilton and Blackstock [5]. The key point is that the use of nonlinear models is recommended, as the presence of shock waves strongly enhances the heat deposition near the focal spot [6].

Depending on the acoustic power and the frequency, cavitation may also occur and strongly influence the lesion shape. It can mechanically damage the tissue, and it locally increases the heating [7, 8]. Cavitation mainly occurs near the focus, where the peak rarefactional pressure is bigger, or at the interfaces between tissues. An elegant work was reported by Chavrier et al. [2] for modeling this effect. The model is incorporated into the acoustic model as a local modification of the attenuation coefficient based on the density of micro-bubbles in the tissue.

It has also been reported in [9] that the nonuniform vibration of the transducer, due to surface waves, can increase the prefocal peak. If prefocal damages are under consideration, it may therefore be worth considering using hydrophone measurements and holographic reconstruction methods to properly predict the acoustic field.

The validation of acoustic simulations in clinically relevant situations is complex as hydrophones are delicate and not designed to work in biological tissues. Hence, validation is often performed in water, using needle hydrophones at low power or fiber optic hydrophones at higher power levels [10–12]. Two studies have also reported measurements respectively in oil [13] and in a gel phantom [9]. These measurement methods result in reliable validation of the numerical schemes. Nevertheless, validating the simulations in such well-controlled media circumvents the modeling of local heterogeneities and the uncertainties on tissue properties.

Many different thermal models have also been proposed during the last decades (see the review of Bhowmik et al. [14]) but the most widely used is Pennes bioheat equation [15]. In this model, perfusion is considered homogeneous in the tissue, and it is therefore ill-suited to the thermal simulations near large blood vessels, especially if the temperature increase is moderate. Detailed discussion about the underlying hypotheses can be found in [16, 17], but, despite strong simplifications, Pennes’ model is generally considered to be accurate enough for a wide range of practical situations.

Thermal damages are usually computed using the thermal dose defined in [18] which is expressed in seconds and represents the equivalent time which would produce the same biological effects at a temperature of 43 °C. Its use for HIFU treatments raises major questions [19], notably its validity above 47 °C, but it is widely used and commonly accepted. A threshold of 14.4×10^3^ s is usually considered for thermal destruction but tissue-dependent thresholds can be found in the review of Yarmolenko et al. [20].

When the temperature in the tissue is high enough, boiling may occur, resulting in the growth of a bubble cloud into the target. It was observed that the presence of boiling bubbles into the tissue induces a high impedance contrast which reflects most of the beam [21]. Therefore the heat deposition pattern is dramatically modified as the energy does not reach the post-focal area anymore. In particular, it is commonly reported that this causes a preferential growth of the lesion towards the transducer. This results in tadpole-shaped lesions for transducers with an *f*-number close to unity of higher [8, 21–23]. Consequently, if boiling occurs during a treatment to be simulated, it is fundamental to consider it in the model.

To date, to the best of our knowledge, no acoustic model enables for simulating the resulting wave scattering. However, equivalent models have been proposed to model the changes in the heat deposition pattern [1, 21]. Wojcik et al. proposed to artificially modify the acoustical impedance of the tissue in the bubbly regions during the acoustical simulations [21]. By contrast, Meaney et al. accounted for the effect of boiling directly in the thermal model by considering that the bubble cloud *shields* the post-focal area [1]. All the energy which would have been deposited after the boiling zone was artificially deposited uniformly over a 0.5-cm-diameter spherical volume centered on the most proximal part of the calculated boiling volume. The resulting lesions were found to be in good agreement with the experimental data.

Based on all these considerations, we designed a complete model to simulate the lesions resulting from HIFU pulses. The acoustical simulations are based on the k-Wave toolbox, and we use a “layer-by-layer” approach to overcome the memory limitations associated with nonlinear 3D simulations. The heat source term calculation is also discussed in terms of energy conservation. A new thermal model, adapted from [1], is then proposed to account for the effect of boiling on heat deposition. Finally, the available experimental data are presented, which include original measurements related to the bubble clouds. Model calibration and validation are presented in the “Results and discussion” section, and the validity domain is subsequently discussed.

## Methods

### Materials

This modeling approach is applied to the particular example of thermal ablations using the Echopulse (Theraclion, Malakoff, France), which is used for the treatment of breast fibroadenomas [24] and thyroid nodules [25]. Benefitting from the important amount and diversity of data collected during preclinical ex vivo and in vivo experiments, the current paper focuses on the modeling of pulses on a rabbit liver.

The acoustic field is generated by a treatment head comprising a single-element therapy transducer and a rectangular confocal imaging probe which enables the monitoring of the treatment using B-mode imaging. The therapy transducer is a spherical cap with a curvature radius of 38 mm and a diameter of 56 mm. The rectangular hole for the imaging probe however breaks the axial symmetry of the generated beam: consequently, three-dimensional simulations are necessary.

Hereinafter, *z* denotes the main propagation axis, pointing from the transducer to the focus, *x* is parallel to the imaging probe, and *y* is orthogonal to it. The frequency used for the treatments is 3 MHz, which corresponds to a wavelength of about 0.5 mm.

At this frequency and with the acoustical powers used in the clinical configuration, which are below 150 W, no cavitation has been detected using a passive cavitation detector. Therefore, the effect of cavitation on the lesion creation is not considered in this work.

### Acoustical simulations

At 3 MHz, considering a 60×60×50 mm domain and the simulation of the nonlinear propagation up to the tenth harmonic with the minimum of two points per wavelength, the pressure field at one given time step in single precision requires more than 40 GB of RAM, making the computations practically intractable on classical hardware.

**Fig. 1:**
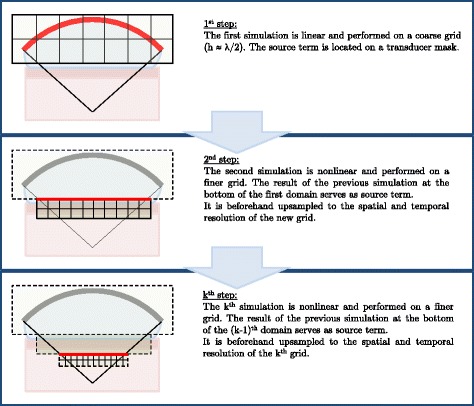
Illustration of the layer-by-layer approach. The spatial and temporal upsampling of the successive source terms is realized in the Fourier domain

Hence, we used a layer-by-layer approach, taking advantage of the beam convergence. Actually, harmonics generation is cumulative, which implies that harmonics are of increasing importance as the beam width decreases. Therefore, we divided the computational grid into a few millimeters thick layers of decreasing width and increasing spatial and temporal resolution (see Fig. 1). The finer grid enabled for the propagation of 10 harmonics which guaranteed a reasonable accuracy according to previous fiber-optic hydrophone measurements in water. The layer-by-layer approach introduces a quasi-one-way approximation which was considered acceptable as we did not want to simulate the imaging system.

**Fig. 2:**
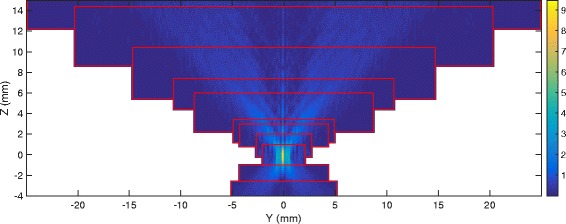
Example of rms pressure field in water simulated using the layer-by-layer approach. Pressure is in MPa. The *red rectangles* represent the limits of the successive simulation domains

All the acoustic simulations were performed using the compiled C version of k-Wave 1.1 [26]. Instead of considering a wave equation, it directly solves the coupled set of equations including mass conservation, momentum conservation and medium constitutive relation using a pseudo-spectral k-space first-order method. In particular, it computes the velocity field, which is convenient for heat source computations. The properties of the different media are listed in Table 1. An example of acoustic field in water is reported in Fig. 2.

**Table 1:** Medium acoustic and thermal properties

	Water	Superficial tissue (skin...)	Liver
Sound speed [ m.*s* ^−1^]	1447	1547	1614
Density [ kg.*m* ^−3^]	1000	1214	996
Attenuation [ dB.c*m* ^−1^.MHz^−*y*^]	2.2×10^−3^	0.62	0.37
*y*	2	1.27	1.27
Specific heat [ J.k*g* ^−1^.*K* ^−1^]	×	×	3700
Thermal conductivity [ W.*m* ^−1^.*K* ^−1^]	×	×	0.55
Perfusion [ *s* ^−1^]	×	×	0.018

As discussed in the “Background” section, the validation is important but complex. In order to confront the k-Wave simulations to experimental data with the real tissues, we excised tissue layers comprising the skin, subcutaneous fat, and muscle from rabbits. They were then maintained on a rigid metallic frame and put into a degassed water tank. The acoustic field through these tissue layers was finally measured at low power using a needle hydrophone and compared to numerical simulations. We found a very good agreement between measurements and simulations over seven biological samples and 55 measurements for different tissue geometry although the biological tissue was modeled as a single homogeneous medium. An article reporting the details of the validation process is currently under review.

### Heat source term

Heat source term is generally computed using the plane-wave approximation. Under the latter hypothesis, the temporal-averaged acoustic intensity can be defined as a scalar quantity:

$$I = \frac{p_{\mathrm{A}}^{2}}{2 \rho_{0} c_{\mathrm{s}}} $$

with *p*_A_ the pressure amplitude, *ρ*_0_ the equilibrium density of the medium, and *c*_s_ the speed of sound. Then, in the linear case, the local expression for the power lost by the wave is

$$Q_{\text{PWlin}} = 2 \alpha_{\text{att}} I $$

with *α*_att_ as the attenuation coefficient of the medium at the considered frequency.

In the general case, the temporal-averaged intensity is defined as

$$\mathbf{I} = < p \mathbf{v} >
$$

with *p* as the pressure field, **v** as the (vectorial) velocity field, and <.> denoting the temporal average over one period. An energy balance over an elementary volume combined with the divergence theorem leads to

$$Q_{\text{exact}} = - \nabla \cdot \mathbf{I} $$

In practical situations, however, when applied to k-Wave simulation results, the numerical computation of the divergence in the last formula using classical finite difference schemes leads to unphysical results, with a locally negative heat source term. Several numerical schemes have been tested, including high-order centered finite difference schemes and derivation in the spectral domain, without solving this issue. However, the cartesian grid is ill-suited to the geometry of our beam, as the angular aperture is greater than $\frac {\pi }{2}$, what might explain these results.

Therefore, as a trade-off, we computed the acoustic power lost by the wave over each *xy* plane as $\frac {dP(z)}{dz} dz$, with:

$${{} {\begin{aligned} P(z) = & \int_{x_{\text{min}}}^{x_{\text{max}}} \int_{y_{\text{min}}}^{y_{\text{max}}} I_{z}(x,y,z) dx\,dy \\& + \int_{y_{\text{min}}}^{y_{\text{max}}}\int_{z_{\text{min}}}^{z} \left(-I_{x}(x_{\text{min}},y,\zeta)+I_{x}(x_{\text{max}},y,\zeta) \right) dy\,d\zeta \\ & + \int_{x_{\text{min}}}^{x_{\text{max}}}\int_{z_{\text{min}}}^{z} \left(-I_{y}(x,y_{\text{min}},\zeta)+I_{y}(x,y_{\text{max}},\zeta) \right) dx\,d\zeta \end{aligned}}} $$

where the two last terms compensate for the power losses through the sides of the computational domain delimited by the planes *x*=*x*_min_, *x*=*x*_max_, *y*=*y*_min_, *y*=*y*_max_, *z*=*z*_min_, and *z*=*z*_max_. The power lost within each voxel layer was then spread according to the repartition of the heat source computed under the linear plane-wave approximation:

$$Q_{\text{cons}}(x,y,z) = \frac{ p_{A}(x,y,z)^{2} }{\int_{x_{\text{min}}}^{x_{\text{max}}} \int_{y_{\text{min}}}^{y_{\text{max}}} p_{A}(x,y,z)^{2} \,dx\,dy} \frac{dP(z)}{dz} dz $$

A comparison with the linear plane-wave approximation is provided in the “Results and discussion” section.

In reality, the energy lost by the wave is partly converted into heat (“absorbed”) and partly scattered by the tissue heterogeneities. Let *α*_abs_ and *α*_scat_, respectively, denote the absorption and scattering coefficients:

$$\alpha_{\text{att}} = \alpha_{\text{abs}} + \alpha_{\text{scat}} $$

Let *A* denote the ratio between absorption and attenuation:

$$A = \frac{\alpha_{\text{abs}}}{\alpha_{\text{att}}} $$

The previously described expressions for the power lost by the wave must therefore be multiplied by *A* in order to compute the heat source term. The value of *A* is of great importance as it is a scaling factor on the heat source. Unfortunately, the values reported in the literature are very variable. For example, Pohlhammer et al. report that *A* is between 0.45 and 0.77 in the liver [27] while Goss et al. report a value of 0.31 [28]. As an uncertainty of factor 2 on the heat source term is not acceptable, *A* must be properly calibrated and not set according to the literature.

### Thermal simulations

In this study, we chose to use the Pennes’ bioheat equation [15] due to its simplicity and the fact that perfusion values are available for most tissues of interest. Moreover, all in vivo pulses were delivered far from the main arteries. The equation was generalized to account for the energy consumed by protein denaturation and boiling based on published data. A simple dependance of the perfusion term with the thermal dose was also implemented. Finally, in the presence of boiling, an equivalent model was used to modify the heat source term: several parameters are introduced which aims at modeling the scattering of the ultrasound beam by the bubble cloud. As they are not directly related to tabulated physical values, they were calibrated. The details of this generalization are described below.

The proposed equation is reported below:

$${{} {\begin{aligned} \rho_{\mathrm{t}} C_{\mathrm{t}}(T) \frac{\partial T(\mathbf{x},t)}{\partial t}\ &= \ k_{\mathrm{t}} \nabla^{2} T(\mathbf{x},t)\ \\&\quad+\ \omega_{\mathrm{b}}(D) \rho_{\mathrm{b}} C_{\mathrm{b}} (T_{\mathrm{b}}-T(\mathbf{x},t))\ +\ Q(\mathbf{x},t) \end{aligned}}} $$

where *T* designates the tissue temperature, *T*_b_ the blood temperature, and *ω*_b_ the *perfusion rate*. *ρ* and *C*, respectively, refer to density and specific heat while the subscripts _t_ and _b_, respectively, refer to tissue and blood. *k*_t_ is the tissue heat conductivity, *D* is the thermal dose in tissue, and *Q* is the heat source term. For the sake of concision, we note **x**=(*x,y,z*) as the dependence in space.

The equation was solved with Matlab using finite differences with a first-order Euler explicit scheme in time and a centered scheme in space.

In order not to overestimate the tissue temperature, we modeled the energy consumed by the protein denaturation and water vaporization using an additional temperature-dependent specific heat.

$$C_{\mathrm{t}}(T) = C_{0} + C_{\text{denat}}(T) + C_{\text{boil}}(T) $$

with *C*_t_ as the temperature-dependent specific heat, *C*_0_=3700 J.kg^−1^.K^−1^, *C*_denat_ as the profile reported in [29], *C*_boil_ as the profile adapted from [30].

The profiles used for *C*_denat_ and *C*_boil_ are based on differential scanning calorimetry (DSC) data. DSC consists in heating a sample of interest and a reference material following a predefined temperature profile. The differences in the power to supply to both samples reveal the reactions occurring in the sample of interest.

To the best of our knowledge, protein denaturation had not been considered in previously reported thermal simulations. However, according to the data reported by Lepock et al. [29] about a rat liver homogenate, it represents 22 kJ.kg^−1^, which corresponds to about 10 % of the energy which is necessary to raise the temperature of the liver from 37 to 85 °C considering a constant specific heat.

For boiling, we considered that the tissue was made up of 75 % of water and that the vaporization was not performed at a constant temperature but progressively, following the profile reported in [30]. As the unit was not specified in the article, we normalized the data as follows:

$$C_{\text{boil}}(T) = \frac{0.75\, H_{\text{vap}}}{\int_{37}^{140} C_{\text{Rama}}(\Theta) d\Theta} \times C_{\text{Rama}}(T) $$

with *H*_vap_=2260 kJ.kg^−1^ as the enthalpy of the vaporization of water, *C*_Rama_ as the profile reported by Ramachandran et al. [30], and *Θ* as the integration variable corresponding to the temperature.

The temperature above which boiling induces additional heating is noted as *T*_boil_ and was set to 85 ° C to be consistant with the specific heat variations used.

When no boiling occurs, we note the heat source term *Q*_0_. It is computed as previously described and convoluted with a 2D Gaussian kernel of standard deviation *σ*_defoc_>0. This convolution aims at modeling the defocusing due to the medium heterogeneity and irregularities in the shape of the interfaces:

$${{} {\begin{aligned} Q(\mathbf{x},t) = Q_{0}(\mathbf{x},t) = A\ Q_{\text{cons}}(x,y,z,t) \otimes \frac{1}{2\pi\sigma_{\text{defoc}}^{2}}e^{-\frac{x^{2}+y^{2}}{2\sigma_{\text{defoc}}^{2}}} \end{aligned}}} $$

where ⊗ denotes the spatial convolution. If needed, the movement of the transducer is modeled by a simple translation of the heat source term, which is responsible for the dependence in time of *Q* and *Q*_cons_.

During the simulations, when the temperature reached *T*_boil_, the heat deposition profile *Q* was modified at each time step.

Let $\mathcal {B}(t)$ denote the set of grid points where *T*>*T*_boil_ at a given time *t*:

$$\mathcal{B}(t) = \left\{\mathbf{x} \mid T(\mathbf{x},t) > T_{\text{boil}} \right\} $$

**Fig. 3:**
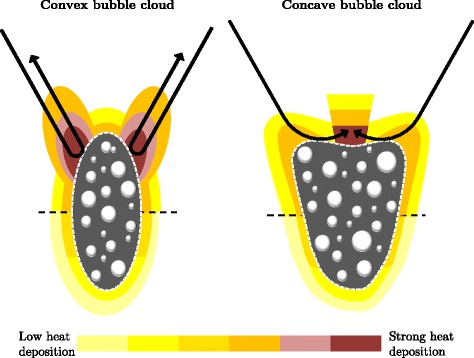
Schematic illustration of the equivalent heat deposition model in the presence of boiling in the *yz* plane. It illustrates the two possible heat deposition patterns for *W*_3_
    This part of the model is probably closely related to our transducer geometry and should not be considered for a generalization. We decided to consider these two different deposition patterns because each of them was not able to separately account for the observed lesion morphology. They were both qualitatively confirmed by k-Wave simulations when changing the properties of the medium around the focal spot to model the presence of different bubble cloud shapes. However, as k-Wave is designed for weakly heterogeneous media, we do not present these simulations. They were only a source of inspiration and do not fully justify our model, which must be considered as an equivalent model.
    The top of the bubble cloud $\mathcal {B}(t)$ was considered *locally convex* at a given time *t* if :
    $${{\kern-17.1pt} {\begin{aligned} \min\left(z\mid(x,y,z)\in\mathcal{B}(t)\right) &= \min\left(z\mid(x,y,z)\in\mathcal{B}(t) \ \text{and}\right.\\ &\qquad\qquad\left.(x,y) = (x_{\text{foc}}(t),y_{\text{foc}}(t))\right) \end{aligned}}} $$
    where the subscript._foc_ refers to the coordinates of the actual focus. Then,
    with $ \mathcal {C}(t) = {\fontsize {9.3pt}{6pt}{} {\begin {aligned}\left \{ (x,y,z) \mid z<z_{\text {foc}} \ \text {and}\ \text {atan}\left (\frac {\sqrt {(x^{2}+y^{2})}}{z}\right)\leq \theta _{\text {cone}}\right \} \end {aligned}}}$

- The heating is null within the bubble cloud itself. This aims at modeling the energy consumed by the additional mechanical and thermal tissue damages induced by the bubble cloud.
  $$\forall \mathbf{x} \in \mathcal{B}(t), Q(\mathbf{x},t)=0 $$
- A shielding coefficient *r*_shield_ is computed as the maximum proportion of the deposited power which is intercepted by the beam within all the *xy* planes:
  $$r_{\text{shield}} = \max\limits_{\zeta}^{} \frac{\sum\limits_{(x,y,\zeta)\in\mathcal{B}}^{}Q(x,y,\zeta)}{\sum\limits_{x=x_{\text{min}}}^{x_{\text{max}}} \sum\limits_{y=y_{\text{min}}}^{y_{\text{max}}} Q(x,y,\zeta)} $$
  Let *z*_shield_ denote the *z* coordinate where the maximum is reached.
- The zone $\mathcal {H}(t)$ where enhanced heating takes place is computed as a morphological dilatation of $\mathcal {B}(t)$ using Matlab *imdilate()* function with a spherical structuring element $\mathcal {S}$ of radius *R*_*SE*_:
  $$\mathcal{H}(t) = \mathcal{B}(t) \oplus \mathcal{S}(R_{SE}) $$
  *R*_*SE*_ is a parameter of the model to be calibrated.
- The total power $P_{\mathcal {H}}$ to be distributed within $\mathcal {H}(t)$ is set as a fraction of the acoustical power reaching the plane *z*_shield_:
  $$P_{\mathcal{H}} = \eta_{\text{intercept}} \, r_{\text{shield}} \, P(z_{\text{shield}}) $$
  with *η*_intercept_ as the coefficient characterizing the efficiency of the power absorption, which has to be calibrated.
- This power is then distributed according to the product of three weighting factors *W*_1_, *W*_2_, and *W*_3_:
  $${{}{\begin{aligned} Q_{\text{boil}}(\mathbf{x},t) = P_{\mathcal{H}}(t) \frac{W_{1}(\mathbf{x},t)\ W_{2}(\mathbf{x},t)\ W_{3}(\mathbf{x},t)}{\sum\limits_{x=x_{\text{min}}}^{x_{\text{max}}} \sum\limits_{y=y_{\text{min}}}^{y_{\text{max}}} \sum\limits_{z=z_{\text{min}}}^{z_{\text{max}}} W_{1}(x,y,z,t)\ W_{2}(x,y,z,t)\ W_{3}(x,y,z,t)} \end{aligned}}} $$
  - *W*_1_ is such that the deposited power decreases as the inverse of the distance to $\mathcal {B}$.
    $${{} {\begin{aligned} \forall h = (x,y,z) \in \mathcal{H}(t), W_{1}(x,y,z,t) = \frac{1}{min\left\{d(h,b) \colon b \in \mathcal{B}(t) \right\}} \end{aligned}}} $$
  - *W*_2_ is such that, beyond *z*_shield_, the deposited power is multiplied by *r*_shield_, which is smaller than 1 and models the shielding due to the bubble cloud.
    $$W_{2}(x,y,z) = \left\{ \begin{array}{ll} 1 & \text{if} \ z\leq z_{\text{shield}} \\ r_{\text{shield}} & \text{if} \ z>z_{\text{shield}} \end{array} \right. $$
  - *W*_3_ is such that, if the top of the bubble cloud $\mathcal {B}$ is locally convex, heating is proportional to the source term in the absence of boiling. If the bubble cloud is locally concave, *W*_3_ is uniform but increased by a factor *W*_+_ within a cone having a *θ*_cone_ angular aperture and the apex at geometrical focus. This intends to model the preferential growing of the bubble cloud towards the transducer. Figure 3 illustrates the two types of deposition patterns, and the implementation details are presented below.

Even if this equivalent model takes complex phenomena into consideration, it only introduces four additional parameters of simple signification:

- *R*_*SE*_, the radius of the zone benefiting from additional heating.
- *η*_intercept_, the proportion of the intercepted power which contributes to the additional heating (the rest is supposed to be simply scattered).
- *W*_+_, the factor characterizing the additional heating due to the refocusing upstream to the bubble cloud when it tends to become concave.
- *θ*_cone_, the angular aperture of the cone where refocusing induces additional heating when the bubble cloud tends to become concave.

In the case where the HIFU transducer is a spherical cap without imaging probe, *W*_+_ and *θ*_cone_ are not necessary, which reduces the number of additional parameters to 2. The calibration and the relative importance of these parameters is assessed in the “Results and discussion” section.

Finally, thermal damages were assessed using the usual thermal dose formula [18]:

$$D(\mathbf{x},t) = {\int_{0}^{t}} 0.5^{43-T(\mathbf{x},\tau)}d\tau $$

where *τ* is the time integration variable. We considered a threshold of 14.4×10^3^ s for tissue destruction. When the thermal dose reached this threshold, we considered that the blood vessels responsible for the perfusion were also coagulated. We modeled this dependence by a simple linear relation:

$$\omega_{\mathrm{b}}(D) = \max\left(1-\frac{D(x,y,z,t)}{14.4\times10^{3}},0\right) \omega_{\mathrm{b}}(0) $$

### Experimental data

During preclinical studies, HIFU pulses of 4 s were performed ex vivo on a bovine liver. After degassing, the samples were immersed in degassed water at 37 °C. One hundred twenty individual pulses were delivered with a median acoustic power of 43.3 W at 3 MHz at a median depth of 16 mm.

The samples were then placed in a cassette which was vacuum sealed in a plastic bag in order for the liver to stick to the cassette walls and immediately frozen at −20 °C overnight. The samples were then sliced orthogonally to *z*, and block-face pictures were taken. Each lesion was then segmented by one operator in order to assess the lesion sizes. Due to the vacuum sealing, the lesion sizes along *z* were overestimated: therefore, we only relied on the lesions dimensions along *x* and *y*. The median boiling onset time was 3 s, and we considered the median lesion sizes over the 40 pulses having a boiling onset time between 2.5 and 3.5 s.

Thirty treatments of 19 pulses were also delivered in vivo on a rabbit liver in the same treatment conditions. The cutting protocol was modified, and no vacuum sealing was performed. Consequently the median size of the global lesion along the three dimensions was considered.

Treatments made up of several 8- and 12-s pulses were also performed in vivo on the rabbit liver with the Echopulse. The pulses were delivered at a median depth of 14 mm with a median acoustic power of 49 W. During these pulses, the focus was describing a circular trajectory in the *xy* plane with diameters of, respectively, 1.1 mm for the 8-s pulses and 1.3 mm for the 12-s pulses.

In order to have three-dimensional representations of the lesions morphology, the liver samples were frozen and sliced orthogonally to *z*. Each slice was subsequently photographed with a qualitatively constant lighting. On the photographs, the unitary pulses were distinguishable and the hypothesis was made that, due to the long cooling time between the pulses (approximately 40 s), the pulses were independent. Therefore, 10 unitary lesions were manually segmented, 5 of which resulting from 8-s pulses and 5 from 12-s pulses. The segmented contours were then stacked to obtain three-dimensional representations of the unitary lesions.

Finally, an archetypal lesion was computed for each pulse type based on the individual 3D reconstructions: the five voxelized lesions of each type were then aligned based on their center of mass and each voxel was retained in the archetypal lesion if it belonged to at least four of the five individual lesions. The resulting 3D volume dimensions were found to be within 200 *μ*m of the median lesion sizes computed over respectively 69 and 72 in vivo pulses, thus validating the representativeness of the segmented lesions.

As boiling occurred during the majority of the pulses, the boiling onset times were estimated based on videos of the B-mode imaging system. The hyperechoic marks (HEM) were also manually segmented right after each 12-s pulse based on the B-mode images. This enabled us to evaluate the growth rate of the bubble cloud itself and to compare it to the simulations.

### Calibration and validation

Firstly, the parameters for which typical values can be found in the literature were set to these values (see Table 1). The grid size was 71×71×111 with a spatial step of 200 *μ*m. The time step was set to 0.05 s. The acoustic power was set to the average power used in the different experiments.

Then, we considered the 4-s pulses and 12 s and tried to defocus the beam in order to obtain a boiling onset time of 3 s during the short pulses and 8 s during the long pulses by modifying the values of *A* (the ratio between absorption and attenuation) and *σ*_defoc_ (characterizing the defocusing).

Finally, the values of *R*_*SE*_, *η*_intercept_, *W*_+_, and *θ*_cone_ were set so that the simulated lesion matched the 12-s lesion.

## Results and discussion

### Heat source term calculation

**Fig. 4:**
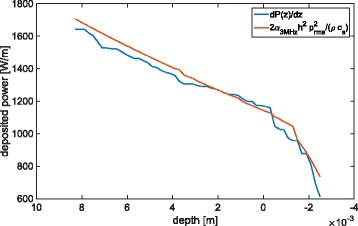
Comparison between plane-wave linear (*red*) and energy-conservation-based (*blue*) heat deposition computations

Figure 4 shows the evolution with *z* of the total deposited power per meter based on the acoustic simulation used for the 4-s pulses. It was calculated as $\int _{x_{\text {min}}}^{x_{\text {max}}} \int _{y_{\text {min}}}^{y_{\text {max}}} Q(x,y,z) dx dy$, with *Q* being computed as described in the “Methods” section for the linear plane-wave approximation and the proposed approach.

It was found that the differences between the two formulas were negligible. The main difference in the profile appears from 4 mm upstream to the focus, where the harmonics become significant and increase the absorbed power with the nonlinear formula. Besides, the curve obtained with the linear approximation is much smoother and less sensitive to the artefactual oscillations arising at the domain boundaries with the layer-by-layer method. Finally, as the difference never exceeds a few percents, the choice of the formula should not have any influence on the simulated lesion. The method we describe could however be more accurate in particular cases where the heating due to nonlinearities becomes predominant, which could for example occur in water in the presence of strong shocks [5].

### Controlability of HIFU lesions in the presence of boiling

For the clinically used 4-s pulses, it must be noted that among hundreds of in vitro and in vivo HIFU pulses, a large proportion of which induced boiling, and no tadpole-shaped lesion was ever observed with the Echopulse. This is probably due to the particular geometry of the transducer which has a large aperture and a hole for the imaging probe. For comparison, the *f*-number of our transducer is 0.68 while other articles report an *f*-number close to unity or bigger: 1.06 [8], 1 [21], 1.79 [22], and 0.98 [23]. Thus, in our case, the ultrasonic wave comes from a wide solid angle to the side of the focal spot and the enhanced heating due to the bubble cloud activity is distributed all around the focus. This probably explains that the lesion does not suddenly grow towards the transducer.

**Fig. 5:**
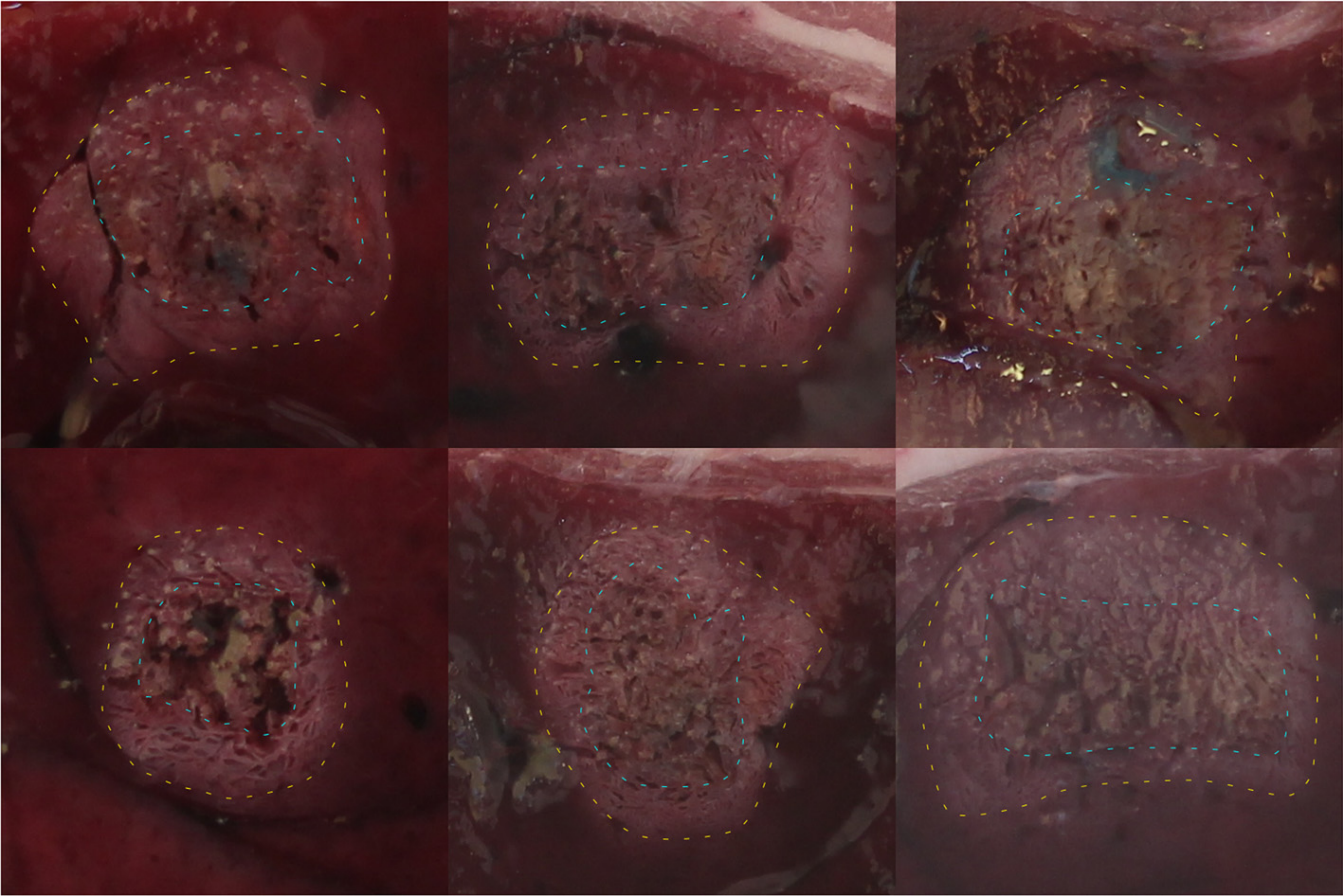
Gross pathology images of lesions. The *yellow dotted line* delineates the global lesion and the *blue dotted line* delineates the overtreated zone corresponding to boiling. The photographs come from six treatments delivered on the livers of six different rabbits with the Echopulse following the clinical procedure

Moreover, the color of the liver tissue is white when usual thermal damages occur but in the presence of boiling, it becomes dark brown [31]. This strongly burned zone never reaches the periphery of the lesion, which shows that boiling is confined to the lesion center (see Fig. 5).

**Fig. 6:**
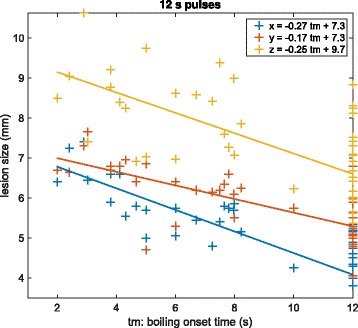
Evolution of the lesion size with boiling onset time for the 12-s pulses. The data come from the in vivo experiments on the rabbit liver. The markers represent the experimental data along *x* (*blue*), *y* (*red*), and *z* (*yellow*). The *lines* are obtained using orthogonal linear regression

Nevertheless, this does not mean that boiling has no influence on heat deposition, especially for longer pulses. As treatments are monitored using B-mode imaging, boiling is clearly visible as hyperechoic marks. It is therefore possible to determine when it occurs. For the 12-s in vivo unitary pulses, we represent in Fig. 6 the size of the lesion versus the boiling onset time.

**Fig. 7:**
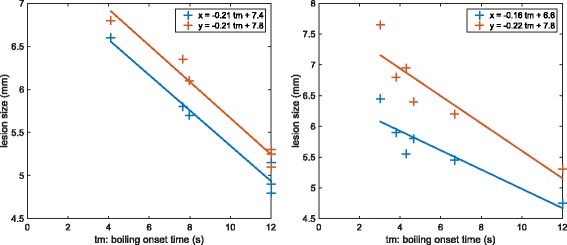
Evolution of the lesion size with boiling onset time for the 12-s pulses for two particular treatments. The data come from the in vivo experiments on rabbit liver. The markers represent the experimental data along *x* (*blue*) and *y* (*red*). The *lines* are obtained using orthogonal linear regression

It clearly appears that boiling favors heat deposition as the lesions are bigger when boiling occurs early. One could argue that boiling is not the cause but a correlated consequence of variations in the power reaching the focus. However, as shown in Fig. 7, this tendency is also observed on single rabbits for neighboring pulses delivered at the same depth. Therefore, slight local defocusing effects are more likely to be responsible for the differences in the boiling time and the in situ power can reasonably be considered as constant, which shows that boiling is responsible for this enhanced heating.

### Thermal simulations

#### Calibration of *A* and *σ*_defoc_

To calibrate these two parameters, we used the boiling onset times for the 4- and the 12-s pulses. The median experimental values were, respectively, 3 and 8.1 s. These experimental data were found to be adequate because they allow for a simple calibration process.

Indeed, the 4-s pulses were delivered at fix positions while the focus was moved by the robotic head during the 12-s pulses. Therefore, increasing *A* increases both boiling onset times but increasing *σ*_defoc_ mainly impacts the 4-s pulses as the energy is already spread by the focus movement during the 12-s pulses.

As a result, these two parameters were set to *A*=0.37 and *σ*_defoc_=290 *μ*m, which resulted in boiling onset times of, respectively, 3 and 7.9 s for the 4- and the 12-s pulses. The value of *A* is higher than the value reported by Goss et al. [28] (0.31) but lower than what is reported by Pohlhammer et al. [27] ([0.45,0.77]). The defocusing induced by *σ*_defoc_ is low, which is consistent with the fact that the experiments were performed in the liver, which is qualitatively homogeneous.

#### Calibration of *R*_*SE*_, *η*_intercept_, *W*_+_, and *θ*_cone_

**Fig. 8:**
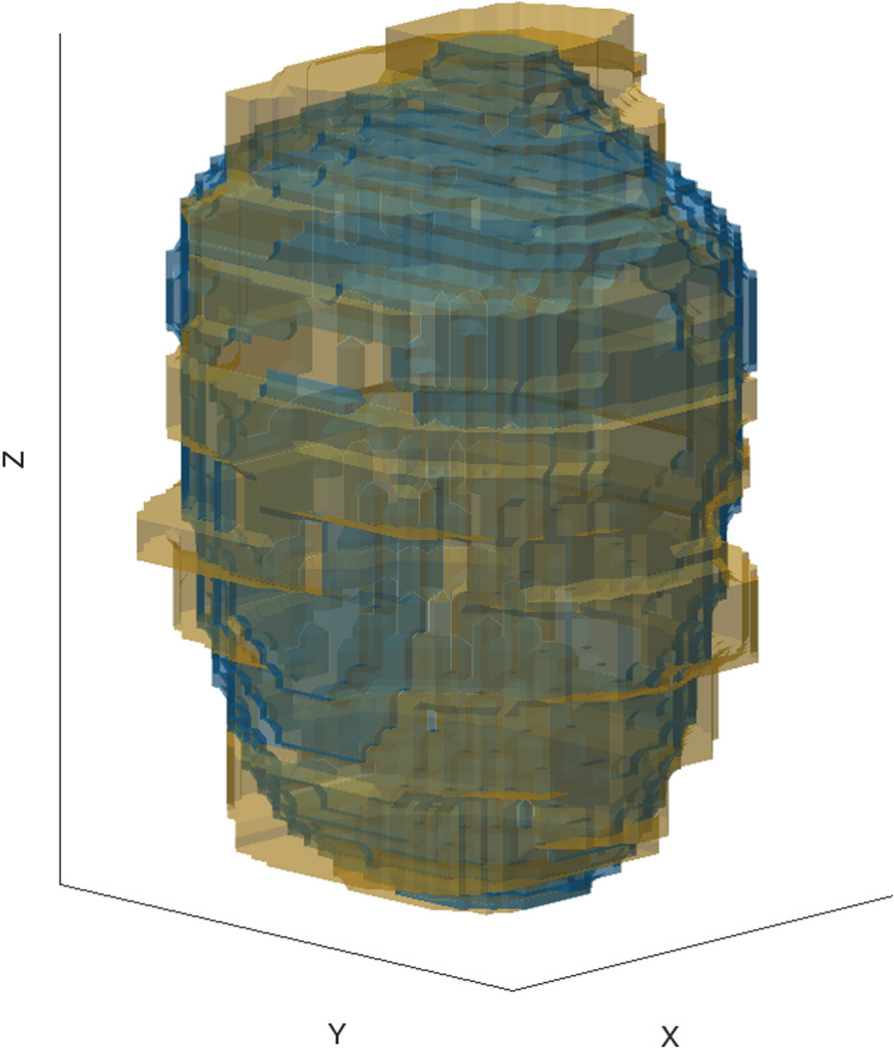
Comparison between the simulated (*blue*) and experimental lesion (*yellow*) for the 12-s pulse. The two lesions are in excellent agreement

The best agreement was obtained for *R*_*SE*_=2.5 mm, *η*_intercept_=0.31, *W*_+_=10, and $\theta _{\text {cone}} = \frac {\pi }{18}$ based on qualitative visual evaluation of the 3D lesions (see Fig. 8).

The influence of *η*_intercept_ is logically very important. The value of 0.31 seems physically acceptable when compared to *A*=0.37: on the one hand, the bubble cloud is generating high frequencies which tend to increase the absorption compared to classical heat deposition but, on the other hand, a proportion of this energy is used to “burn” the tissue and to mechanically damage it.

The influence of *R*_*SE*_ on the final lesion is not very important although it has a significant impact on the computational time. As the additional power due to boiling decreases linearly with the distance to the boiling zone, increasing *R*_*SE*_ above this value simply leads to consider grid points for which the additional heating is negligible. Decreasing *R*_*SE*_ leads to smaller values of the coefficient *η*_intercept_ and is physically less plausible.

Increasing *W*_+_ increases the frequency of the shifting between the two heating modes described in Fig. 3. When *W*_+_ is lower, the bubble cloud can become visually concave, which has not been observed on the experimental B-mode images. In addition, *W*_+_=10 enables for properly reproducing the height of the 12-s lesion.

*θ*_cone_ must be small in order to model a refocusing above the bubble cloud. It must be noted that, for classical spherical transducers without imaging probe, the bubble cloud is very unlikely to become concave. Therefore, the expression of *W*_3_ can be restricted to the case of the locally convex bubble cloud, thus eliminating the need for *W*_+_ and *θ*_cone_.

All calibration data are reported in Table 2.

**Table 2:** Calibration data

	4-s pulses: unitary lesion sizes [ mm]			Boiling onset time [ s]	
	*x*	*y* *z*	4-s pulses	12-s pulses	
Measurements	2.55	2.65 6	3	8.1	
Simulations	2.4	2.8 4.2	3	7.9	

#### Evolution of the lesion size with boiling onset time

To evaluate if the influence of boiling was properly modeled, we performed a linear regression on the lesions sizes with respect to the boiling onset times (see Fig. 6). The boiling onset time is noted *tm* for *time to mark* as it was detected based on hyperechoic marks on the B-mode images. The resulting slopes are expressed in mm s^−1^ and, as boiling tends to increase the efficiency of the heat deposition, they are negative: the later the boiling, the smaller the lesion. When the modulus of the slope is big, it means that boiling has a strong influence on the lesion size along this dimension.

Boiling onset time estimation was subject to uncertainties of the order of 0.1 s, and the precision on the lesion sizes was of the order of 0.1 mm; therefore, we performed orthogonal linear regressions. A classical linear regression would have lead to errors of more than 50 % on the slopes, which highlights the importance of a proper analysis of the data in the context of uncertainties.

For the 12-s pulse simulations, the slopes characterizing the evolution of the lesion sizes along *x*, *y*, and *z* with the boiling onset time were, respectively, −0.21, −0.27, and −0.63 mm s^−1^. The slopes obtained by regression on the experimental data were, respectively, −0.27, −0.17, and −0.25 mm s^−1^. These data are reported in Table 3 with 90 % confidence intervals.

**Table 3:** Validation data: slope of lesion size vs boiling onset time in mm/s for the 12-s pulses. Slopes obtained by orthogonal linear regression on the experimental data are represented by the best fit and the 90 % confidence interval

	Slope of lesion size vs boiling onset time [ mm/s]		
	Along *x*	Along *y*	Along *z*
Regression on experimental data	−0.27 [ −0.34; −0.15]	−0.17 [ −0.27; −0.1]	−0.25 [ −0.4; −0.03]
Simulations	−0.21	−0.27	−0.63

Along *x* and *y*, the slopes computed based on the model are in good agreement with the experiments as they are within the 90 % confidence interval. The experimental dependence of the size along *z* on boiling onset time is found to be smaller in the measurements than what was predicted by the model. The value derived from the model could be reduced by setting a smaller value for the coefficient *W*_+_, but this discrepancy probably highlights the limit of the equivalent model. In general, the influence of boiling appears more complex to model along the *z* axis.

For the 4-s pulse simulations, the slopes computed based on regressions on the experimental sizes along *x* and *y* were, respectively, −0.5 and −0.6 mm s^−1^. The slopes obtained based on the simulations were, respectively, −0.6 and −0.7 mm s^−1^ (see Table 4). The values derived from the simulations were therefore in good agreement with the slopes estimated by regression on the experimental data. As described in the “Methods” section, the values along *z* were not considered for those pulses due to the slicing method which biased the sizes along *z*.

**Table 4:** Validation data: slope of lesion size vs boiling onset time in mm/s for the 4-s pulses. Experimental data along *z* were not considered due to the bias introduced by the slicing method

	Slope of lesion size vs boiling onset time [ mm/s]	
	Along *x*	Along *y*
Regression on		
experimental data	−0.5	−0.6
Simulations	−0.6	−0.7

#### Growth of the bubble cloud for the 12-s pulses

**Fig. 9:**
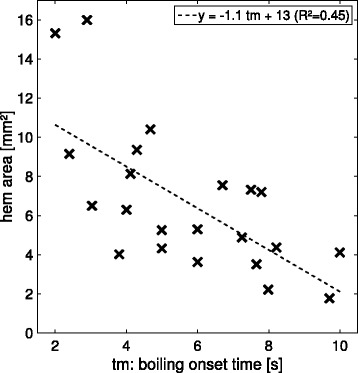
Change of the final HEM area as a function of boiling onset time. HEM areas were assessed in the *xz* plane based on the manual segmentation of the B-mode images captured before and after each pulse in the context of in vivo experiments on rabbit liver

With these parameters, the final area of the bubble cloud in the *xz* plane for the 12-s pulses is 5 mm^2^, which equals the median experimental value evaluated based on a manual segmentation of the B-mode images. As for the lesion sizes, we also performed a linear regression on the bubble cloud area with respect to the boiling onset time. The slope was negative as, logically, the later boiling occurs, the smaller is the bubble cloud area. The estimated slope was −1.1 mm^2^ s^−1^ (see Fig. 9). By comparison, the slope obtained from simulated data was −1.2 mm^2^ s^−1^. This further validates the fact that our equivalent model is relevant to simulate the influence of boiling on the heat deposition pattern.

#### 8-s pulses and groups of 4-s pulses

**Fig. 10:**
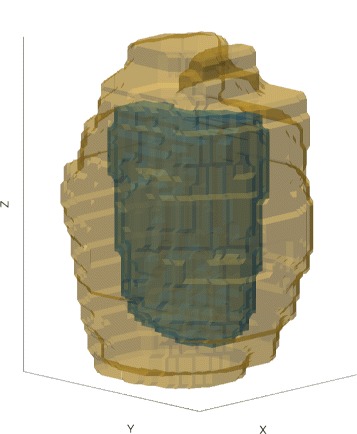
Comparison between the simulated (*blue*) and experimental lesion (*yellow*) for the 8-s pulse. The poor agreement between the two lesions can be explained by the fact that the segmented unitary lesions were not independent from each other

Despite the good agreement between simulated and experimental lesions for the 4- and 12-s pulses, we denoted a poor agreement for the 8-s pulses. As shown on Fig. 10, the simulated lesion are significantly smaller than the segmented lesions. These discrepancies are likely to be due to the fact that the segmented lesions do not come from unitary pulses for the 8 and 12 s. In the case of the 8-s pulses, the spacing between two adjacent lesions was 4 mm instead of 5 mm for the 12-s pulses (see Table 5). This resulted in a greater overlap between neighboring lesions. Consequently, although they could be distinguished one from another, each pulse may have benefited from additional heating due to the presence of the bubble cloud created by the previous pulse. This hypothesis has been qualitatively confirmed by the retrospective visualization of captured movies acquired by the B-mode imaging system. In addition, the median inter-pulse cooling time was 48 s for the 12-s pulses and only 39 s in median for the 8-s pulses which could reinforce the influence of the adjacent pulses.

**Table 5:** Experimental conditions related to the influence of neighboring pulses

	Median cooling	Spacing along	Spacing along	Overlap
	time (s)	*x* (mm)	*y* (mm)	(%)
4-s pulses	22	1.8	1.6	45
8-s pulses	39	4	3.5	19
12-s pulses	48	5	4.3	6.7

The overlap is calculated based on elliptic representations of the lesions in the *xy* plane. It is calculated as the ratio between the area which is covered by at least two individual lesions and the area covered by the global lesion

The same phenomenon occurs for groups of 4-s pulses if we consider groups of 19 pulses with an important overlap. Even when these groups of 19 pulses were simulated including relevant cooling time, the height of the simulated lesion was 6 mm, which is smaller than the 9 mm experimentally measured. The thermal build-up, which was accounted for in the simulations, is therefore not the only factor which increases the size of adjacent lesions.

#### Validity domain

The last observations highlight the main limit of the proposed model. It is efficient for simulating unitary pulses but, in the context of whole treatments, it does not account for the additional heating due to remaining bubble clouds created by the previous pulses.

It must also be pointed out that the model has been specifically designed for the studied case. In the field of HIFU thermal ablation, the transducer geometry and treatment parameters can greatly vary depending on the clinical application: therefore, some phenomena which are not modeled here can be of significant importance for other devices. For instance, it has been shown that increasing the *f*-number of the transducer increases the influence of thermal lensing [32]: as our transducer had a low *f*-number of 0.68, it was considered to be negligible in our case.

The modifications of the tissue properties with the thermal dose were not modeled either, except for perfusion. Therefore, the lesion resulting from several pulses delivered at the same location would probably not be properly modeled.

More importantly, this model has been developed based on pulses delivered on liver, while the Echopulse is designed to treat breast fibroadenomas and thyroid nodules. Validating the simulations in several different animal tissues could however be relevant to evaluate the potential differences in lesion creation.

## Conclusions

In this study, important issues regarding the modeling of HIFU thermal ablations are discussed. A complete numerical model of HIFU pulses was designed and carefully validated based on experimental data. Its calibration was thoroughly discussed, and we described a simple method to estimate the ratio between absorption and attenuation and the defocusing related to the medium heterogeneity.

An equivalent model, adapted from [1], was proposed to simulate the modified heat deposition pattern in the presence of boiling. First, we showed that boiling did not make the lesion sizes unpredictable with our transducer. Then, in order to fit the experimental lesion shape, it was found that the influence of boiling on the heat deposition was complex and could be modeled as an alternation between two regimes. The influence of the remaining bubbles clouds in the context of adjacent pulses was also highlighted. Thus, beyond the predictive capability of the equivalent model, which is by nature limited, this article illustrates the use of numerical simulation as a tool to confront our understanding of the lesion creation process to real data.

Moreover, the equivalent model was validated against cheap and usually unexploited experimental data including the evolution of bubble clouds areas evaluated on B-mode images and the slope characterizing the evolution of the lesion sizes with the boiling onset time. Therefore, this study is also intended to encourage the creative use of experimental data to stimulate the design of complete models of HIFU treatments, from the acoustic beam to the induced lesions.

Future works should include modeling the additional heating due to previously created bubble clouds in the context of whole treatments. Our approach could also benefit from more fundamental experiments aiming at measuring the acoustic field scattered by the bubble cloud.

## Ethics, consent, and permissions

The herein reported in vivo experiments have been approved by the ComEth ANSES/ENVA/UPEC ethics committee with the reference number 2015030416468883.
